# On the Path towards a “Greener” EU: A Mini Review on Flax (*Linum usitatissimum* L.) as a Case Study

**DOI:** 10.3390/plants12051102

**Published:** 2023-03-01

**Authors:** Panteleimon Stavropoulos, Antonios Mavroeidis, George Papadopoulos, Ioannis Roussis, Dimitrios Bilalis, Ioanna Kakabouki

**Affiliations:** Laboratory of Agronomy, Department of Crop Science, Agricultural University of Athens, 11855 Athens, Greece

**Keywords:** European Green Deal, Farm to Fork, flax, linseed, Sustainable Development Goals

## Abstract

Due to the pressures imposed by climate change, the European Union (EU) has been forced to design several initiatives (the Common Agricultural Policy, the European Green Deal, Farm to Fork) to tackle the climate crisis and ensure food security. Through these initiatives, the EU aspires to mitigate the adverse effects of the climate crisis and achieve collective prosperity for humans, animals, and the environment. The adoption or promotion of crops that would facilitate the attaining of these objectives is naturally of high importance. Flax (*Linum usitatissimum* L.) is a multipurpose crop with many applications in the industrial, health, and agri-food sectors. This crop is mainly grown for its fibers or its seed and has recently gained increasing attention. The literature suggests that flax can be grown in several parts of the EU, and potentially has a relatively low environmental impact. The aim of the present review is to: (i) briefly present the uses, needs, and utility of this crop and, (ii) assess its potential within the EU by taking into account the sustainability goals the EU has set via its current policies.

## 1. Introduction

**Figure 1:**
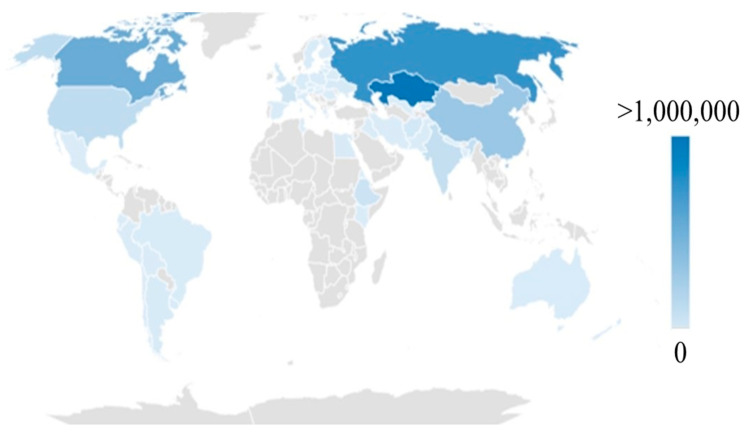
Global production of flaxseed in 2020. In the color scale of the figure the light gray areas correspond to 0 tons whilst dark blue ones correspond to over 1,000,000 tons of produced linseed. Data retrieved from https://www.fao.org/faostat/en/#data/QCL (accessed on 10 January 2023) [1].

Today, flax is grown in more than 50 countries around the globe [1], with Canada, India, Russia, Kazakhstan, and China being some of the major producers [2,3] (Figure 1). In 2020, Canada and France were the two biggest exporters of flaxseed and flax fiber, respectively [4]. It has been estimated that the market of flax is expanding rapidly [5,6], indicating renewed interest, possibly attributed to recent research developments [7], as well as the recognition of the multiple applications of flax [8]. Concurrently, crops that could contribute to climate change mitigation by reducing the environmental impact of agriculture are under the spotlight [9]. The degradation of the environment (amongst others) has led the United Nations to deliver a set of Sustainable Development Goals that aim to achieve collective prosperity for both people and the planet [10]. In the European Union, the Common Agricultural Policy (CAP), the European Green Deal (EDG), and Farm to Fork (F2F) aim to achieve the same goals [11,12,13]. Policymakers, researchers, and governing bodies across the EU are constantly searching for smart solutions to the everlasting problems of climate change and food insecurity. The utilization of resourceful crops could be a viable answer [14]. The present review aims to concisely present the potential of flax and evaluate whether or not it constitutes a crop that could facilitate the implementation of current European agricultural policies and strategies.

## 2. Uses and Applications

Flax is a multipurpose crop. In fact, the Latin term “*usitatissimum*” in the scientific name of flax translates as “the most useful” one, due to its multiple uses [15]. The two main products of the crop are its seeds and its fibers. The fibers have many applications in the textile industry and are considered rather strong, mainly due to their high cellulose content [16,17]. The mechanical and physical properties of flax fibers are presented in Table 1. From dressing fabrics and bed sheeting to twine and ropes, the fibers of flax have been heavily utilized for industrial purposes [16]. Some of the highest-quality textiles such as damasks and lace are made from the fibers of flax (also known as linen) [16]. The fibers are also utilized in the paper industry and, interestingly, they have been used in the printing of banknotes [16]. They can be used to reinforce recycled paper, improving its strength, in the production of insulation batts instead of glass fibers, the production of wound dressing cloths [18], and the production of geotextiles [19]. Recently, the automotive industry started utilizing flax fibers as an eco-friendly source of composite materials [20].

The seeds of flax are rich in bioactive substances such as alpha-linolenic acid (ALA), proteins and lignan, rendering them as a nutritious source of human and animal food [21,22] (Table 2). Flaxseeds can be consumed as whole seeds or milled powder [23] and are rich (~40%) in oil [24]. The oil is edible and very nutritious [25,26] (Table 3) and has a pleasant taste and aroma [27]. It also has a high content of linolenic acid (48.5–68.5%), a low content of saturated fatty acids, and is rich in ω-3 and ω-6 [27]. According to Madhusudhan [28] and Bilalis et al. [29], flax oil is amongst the best sources of omega-3- fatty acids and perhaps the richest source of a-linolenic acid.

Flax oil is rich in bioactive compounds that can potentially prevent inflammation, hormonal disorders, cardiovascular diseases, infections, bone diseases, cancer, and many more [30,31] by modulating several signaling pathways [32]. Besides their suggested application in the prevention of disorders, flaxseeds and flax oil have also been found to possess remedial properties [23]. Patients with peripheral artery disease [33], cardiovascular diseases [34], and hemodialysis patients with dyslipidemia [35] have reportedly been found to have a positive response to the consumption of flaxseed. Parikh et al. [36] suggested that the consumption of flaxseed might alleviate arrhythmias and conditions that may result in heart dysfunction. Similarly, Tang et al. [23] stated that the consumption of flax-based food products could help patients with diabetes. The phenolic compounds of flax have also been proposed as possible antibiotic alternatives [18]. Fatty acids such as ω-3 and ω-6, which are major components of flaxseed oil, can be used in cosmetics [37] as they improve the health of hair and have been associated with the regeneration of skin tissues [27].

**Table 2:** Flax seed nutritional value per 100 g of seed. Data retrieved from https://fdc.nal.usda.gov/fdc-app.html#/food-details/169414/nutrients (accessed on 10 January 2023) [38].

Content	Amount	Unit
Water	6.96	g
Energy	534	kcal
Protein	18.3	g
Total sugars	1.55	g
Carbohydrates	28.9	g
Total lipids	42.2	g
Total dietary fibers	27.3	g
Ash	3.72	g
Vitamin B-6	0.473	mg
Vitamin C	0.6	mg
Vitamin E	0.31	mg
Vitamin K	4.3	μg
Ca	255	mg
Cu	1.22	mg
Fe	5.73	mg
K	813	mg
Mg	392	mg
Mn	2.48	mg
Na	30	mg
P	642	mg
Se	25.4	μg
Zn	4.34	mg

Besides its seeds and oil, it should also be mentioned that flax sprouts and microgreens have been proven to be highly nutritious [39]. The consumption of sprouts (young seedlings of freshly germinated seeds) and microgreens (young seedlings that have developed their first true leaves) has recently attracted increasing attention [40]. According to some studies, flax sprouts and microgreens are richer in essential micronutrients (such as Fe, Mn, and Zn) and have higher antioxidant capacity compared to the seeds [40,41], and are rich in water-soluble proteins, free amino acids, and free fatty acids [42].

According to Liu et al. [43], the inclusion of linseed (seeds and/or seed oil) in the rations of goats can regulate their blood lipid content. Similarly, Brito and Zang [44] concluded that flaxseeds could be beneficial to the health of dairy cows. Weston et al. [45] found that flaxseed consumption increases the lifespan and improves the liver function in hens, and Popescu et al. [46] noted an enhancement in the intestinal health of flaxseed-fed chicken. Based on the findings of Neelley and Herthal [47], feeds that include flax can reduce the chances of laminitis in horses, while Ngcobo et al. [48] reported that the consumption of flax could improve the semen quality of livestock. The high nutritional value of flax not only benefits the health of productive animals but also the quality of the meat, dairy, and eggs they produce, thereby improving the quality of value-added animal products [49]. For instance, the quality of pork meat was improved in flax fed pigs [50] and the quality of eggs was improved in hens [51]. These findings, alongside the nutritional value of flax seeds, can also be perceived as a potential enhancement of food security (FS). In order to achieve FS, food must not only be adequate but also nutritious [52]. This aspect of FS is now more relevant than ever, as the rising anthropogenic CO_2_ emissions have been suggested to negatively affect the nutrient content of major food crops including rice, potato, wheat, maze, and barley [53,54,55]. According to the literature, high CO_2_ atmospheric levels could instigate micro, macro, and protein deficiencies [53,54,55]. Interestingly, in a study by Hacisalihoglu and Armstrong [56], the authors evaluated several flax varieties and distinguished six of them (Omega, Clli1374, Clli1418, Clli1821, Clli643, and Clli2033) as superior due to their high nutritional content under elevated CO_2_ stress.

**Table 3:** Flax oil nutritional value per 100 g of oil. Data retrieved from https://fdc.nal.usda.gov/fdc-app.html#/food-details/1103860/nutrients (accessed on 10 January 2023) [57].

Content	Amount	Unit
Water	0.12	g
Enegy	884	kcal
Protein	0.11	g
Total lipids	99.98	g
Vitamin E	0.47	mg
Vitamin K	9.3	μg
Saturatred fatty acids	8.98	g
Linoleic acid	16	g
Alpha linolenic acid	57	g
Trans fatty acids	0.094	g

Lastly, the profile of flaxseed oil makes it suitable for biodiesel production [58]. Even though flaxseed-based biofuels have a lower energy potential (48.8 MJ/kg) in comparison to fossil diesel fuels (57.14 MJ/kg) [59], the use of plant based-fuels can reduce the emissions of greenhouse gases and, in the near future, facilitate climate neutralization. The stems of the plant can also be turned into pellets for energy production [60]. Other uses include phytoremediation applications [61], biochar production [62], as a nematicide [63], and as a food preservative in the food industry [18,64].

## 3. Crop Needs and Management

The fertilization needs of flax have been the subject of several studies. Based on the literature, flax responds positively to the application of nitrogen (N) fertilization as it promotes vegetative growth, canopy development and structure, and improves the yield [65,66,67]. Unsurprisingly, a wide range of N fertilization rates (20–150 kg N per ha) have been tested and proposed for flax in the literature [66,67]. Of course, the optimal level of N fertilization also depends on the soil properties and the cultivar [66]. Excessive use of N fertilization in fiber flax has been proven to negatively affect the yield as the prolongation of the vegetative phase results to greater susceptibility lodging and diseases [68]. Notably, in a study by Herzog et al. [65], the authors concluded that N fertilization is negatively correlated with the quality of flaxseed, as increasing N fertilization rates reduced the a-linoleic acid content in linseed oil. On the contrary, balanced phosphorus (P) fertilization can improve the quality traits of flax seed oil [69]. Moreover, P fertilization has been found to improve the dry matter accumulation, the yield (both in seed and in fiber), and the yield components of flax [69,70,71,72,73]. Similarly, the application of potassium (K) fertilization can increase the biomass of the plants and the grain yield [74,75], although according to Berti et al. [76], K fertilization (as well as P) does not affect the content, composition, and yield of flax oil. In a review by Cui et al. [67], the authors concluded that the application of organic fertilizers in flax significantly improves the quality of the grains, but its effects on the yield were in doubt. In the same study, the authors recommended (based on the literature) that the optimal fertilization in flax ranges among 75–150 kg N/ha, 35–75 kg P_2_O_5_/ha, and 35–52.5 kgK_2_O/ha. The combined application of organic and inorganic fertilization has also led to promising results as it improves the agronomic traits of the crop whilst maintaining soil fertility and enhancing the efficiency of the fertilizers [77,78].

Similar to fertilization, the irrigation needs of the crop are dependent to the climate, the soil properties, and the cultivar [79], thereby a wide range of drought tolerance has been reported in flax [80]. Several studies highlight the importance of irrigation, as the short root length of flax often prevents it from reaching deep underground water [81,82], and water insufficiency can negatively affect the agronomic traits and the yield of flax [83,84,85]. Other studies suggest that flax could be regarded as a drought tolerant crop, and its tolerance could partially be attributed to proline, which can regulate cell osmosis [86]. The water needs of flax in the literature vary vastly. Case in point, according to Singh et al. [87], 450–750 mm of water per season is sufficient for flaxseed in India, yet in a study by Kakabouki et al. [88], in Greece, less than 400 mm of water is enough for the crop to perform well. Overall, fiber flax cultivars are characterized by higher water needs compared to flaxseed cultivars [89]. Interestingly, N fertilization can affect the crop’s irrigation needs and mitigate drought stress [90,91]. This finding is in accordance with the work of Rajabi-Khamseh et al. [92], who observed that the use of plant-growth promoting microorganisms (PGPM) had a positive effect on the yields of flax and mitigated drought stress. 

It should be noted that the literature suggests that PGPMs can improve the nutritional status of plants and the availability of N [92]. Thingstrup et al. [93] concluded that the presence of arbuscular mycorrhiza fungi (AMF) is crucial for flax when the soil P levels are below 40 mg P per kg of soil. Similarly, Rahimzadeh and Pirzad [94] found that AMF and phosphate solubilizing bacteria can improve the performance of the crop and the quality of flaxseed. Studies conducted regarding the efficiency of PGPM-based biofertilizers in flax have reported promising results both quantitatively and qualitatively [95,96]. As the agri-environmental policies of the EU aspire to reduce chemical inputs in agriculture and promote organic farming [12], their potential could be of great significance. However, and despite the fact that PGPM-based products are often considered cost effective, environmental-friendly, and organic compliant [97], the complexity of their production process and their practicality [98] are disadvantages that have not yet been fully addressed and require further research. 

PGPMs have also been proven to protect flax from pest infestations [99]. Even though several pests [100] and diseases [101] have been reported in flax, weeds pose arguably the greatest threat to the yield [102]. Flax is characterized as a poor weed competitor [103], mainly due to its slow growth rate during the early growth stages [104]. Based on the literature, severe weed infestations can significantly reduce the yields of both flaxseed and fiber flax, or even result in complete crop failure [105]. Chemical management constitutes the most well-adopted weed management practice; however, as the extensive use of herbicides has been correlated with the degradation of the environment [12], several alternative weed management strategies have been studied and/or proposed for flax. These strategies include the selection of more competing cultivars [102], altering the seeding rate or date [102,106], crop rotations [107], and the use of mulches [108]. These alternatives alongside the use of organic fertilizers and biopesticides [109] also enable flax to be grown organically. Notably, the performance of flax has been reportedly improved in organic systems under no-till regimes [29,107].

## 4. Flax in the Era of “Green” Policies

**Figure 2:**
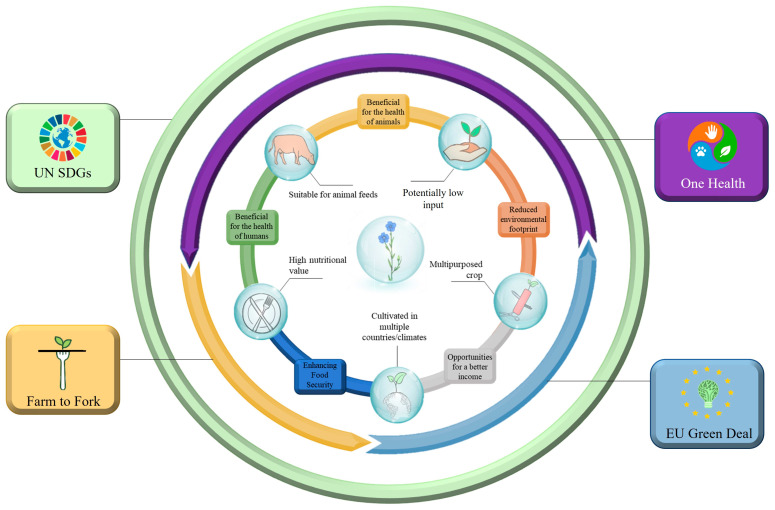
The benefits that flax offers to the environment, the humans, and the livestock. These benefits are depicted under the context of the current agricultural policies that the EU has committed to.

Before we investigate the compatibility of cultivating flax with the goals of the European Union (EU), we first need to define these goals. Recently, the EU has launched the Common Agricultural Policy (CAP) 2023–2027 [110], has committed to the United Nations Sustainable Development Goals (SDGs) [10], and aspires to implement the European Green Deal (EDG), and Farm to Fork (F2F) [12,13]. All these policies/initiatives set their own objectives, with most of them being consensually oriented or even overlapping (e.g., all of them emphasize the importance of sustainability). Here, we propose an easy and quick method to describe and organize their “common ground” by following the One Health (OH) paradigm. According to the World Health Organization, the OH can briefly be described as the effort to “optimize the health of people, animals and the environment” [111]. Therefore, we will evaluate flax based on how it benefits the environment, the well-being of humans, and the health of the livestock (Figure 2).

Most of the applications of flax on human and animal health have been discussed in the Section 2. The environmental benefits can be assessed based on the crop’s carbon (CO_2_^−^_eq_ emitted per kg of product) and water (m^3^ required per kg of product) footprints. As elaborated above, flax is cultivated in several countries and areas, on soils with a wide range of different properties, and under different climatic conditions. As expected, the literature is filled with contrasting findings when estimating the CO_2_^−^_eq_ per kg of flax fiber or seed. Case in point, according to Niels de Beus et al. [112], the carbon footprint of flax fiber is approximately 0.9 kg CO_2_^−^_eq_ kg^−1^, while Dissanayake et al. [113] reported it at over 11 kg CO_2_^−^_eq_ kg^−1^. Niels de Beus et al. [112] concluded that the fertilization is the most influential factor in the quantification of carbon footprint, thus the wide range of results is understandable. However, and despite of the inconclusive CO_2_^−^_eq_ estimations in the literature, it is possible to compare the climate impact of flax with that of other fiber crops. Cotton is arguably regarded as the major natural fiber crop around the world [114], and in the EU, its fibers are widely used in the textile industry [115]. According to a report by Sadin and Ross [116], on average, the carbon footprint of cotton (0.5–4 kg CO_2_^−^_eq_ per kg of fibers) could even be as much as four times higher than that of flax (0–0.8 kg CO_2_^−^_eq_ per kg of fibers). Similarly, it has been reported that the average CO_2_^−^_eq_ per kg of flaxseed oil is less than half that of sunflower oil [117]. Regarding the water footprint of flax fibers, according to a report by the Institute for Water Education (IWE) of UNESCO, producing 1 kg of them requires roughly three times less water, compared to cotton fibers [118]. Admittedly, the water footprint (m^3^ of water per kg of product) of the flax seeds and flaxseed oil is not promising, as the IWE report found that it is 0.6–2 times higher than that of the three most important seed oil crops of the EU: rapeseed, sunflower, and soya (and their respective seed oils) [119]. However, it should be noted that this study was conducted based on global data during 1996–2005, and that the significantly higher water per seed kg of flax was mainly attributed to a significantly higher green water footprint. For instance, the green water footprint of flaxseed and rapeseed oil was estimated at 8618 and 3226 m^3^ per seed tones (respectively), whilst the blue water footprint of the two oils was estimated at 488 and 438 m^3^ per seed tones (respectively) [118]. Therefore, perhaps the findings of this report could be partially unindicative or outdated.

Moreover, the financial potential of flax should also be considered. Afterall, flax is regarded as a cash crop that generates a good revenue in several parts of the world [8]. According to a recent report on the global flax market insights, the seed market that currently sits at over 400 million USD is expected to reach approximately 725.9 million USD by 2028 [6]. Likewise, the linen fabric industry is expected to slowly but steadily expand (the Compound annual growth rate is estimated to increase at least by 3% by 2029), and the EU is estimated to hold, if not the biggest, one of the biggest shares of the market [5]. One of Europe’s (and the UNs’) main objectives is the eradication of poverty [10]. A significant portion of unprivileged people are located in rural areas and often their main income derives from agricultural activities [120]. Therefore, it is crucial to provide farmers with alternatives that could improve their income, whilst tackling the degradation of the environment. At a farm level, the adoption of dual-purpose (DP) cultivars could increase the profits of farmers [121]. Even though the majority of the seed oil varieties are not suitable for fiber extraction, some varieties can sufficiently yield fiber as a by-product [122]. In some studies, the yields of such cultivars have been recorded to reach up to approximately 1000 and 2000 kg of fiber and oil, respectively, per ha [122]. Via the utilization of DP flax cultivars, it is possible to exploit the crop to its full financial potential. Shaikh et al. [123] proposed that the woody straws of DP flax, which are essentially by-products of the scutching process, can be used in the production of low-cost paper. Similarly, according to Papadopoulos and Hague [124], flax shives can be used in the production of particleboards. Therefore, it is possible to reduce agro-waste materials that in many parts of the EU are disposed through burning (despite of the strict regulations that prohibit this practice [125]) whilst simultaneously providing additional revenue to farmers.

The EU has recognized the potential of flax. In a recent study by the European Parliamentary Research Service (EPRS) on the future of crop protection in the Union, the authors proposed flax as a resilient crop that can acclimatize and could be adopted by farmers all across the EU [126]. Notably, the authors regarded flax as an oil crop that could be introduced in lieu of other major crops that are more susceptible to biotic stress (pests and diseases). Interestingly enough, this is the definition of alternative crops (ACs), as it was proposed by Isleib [127]. In fact, in their study, EPRS mentioned flax as an AC. Provided that flax could indeed be considered as an AC in the EU, this would add more to its value as a crop. According to the literature ACs have been proposed as a versatile tool that could facilitate the implementation of the EGD and simultaneously enhance Food Security [14,128]. Nonetheless, the introduction (or re-introduction in the case of retroactive crops) of ACs has its limitations [129]. In most cases, ACs are characterized by limited information on the proper cultivation practices, lackluster market presence, and few available cultivars [129]. Of course, this is not the case with flax. As mentioned above, the flax market is expanding, the literature thrives with information regarding its cultivation, and there are more than 120 registered cultivars in the European common catalogue of varieties of agricultural plant species [130]. Moreover, due to the limited research focusing on ACs, they are usually excluded from policymaking [129]. However, this is hardly the case with flax. On the other hand, the unadaptable attitude of farmers could be an obstacle. Studies have found that farmers are often reluctant to adopt an AC, partially due to limited knowledge or information [131]. The perceptions and attitudes of farmers have also been found to correlate with their education and age [132]. However, the Commission has already included generational renewal in its action plans, aiming to attract young and educated farmers and entrepreneurs in rural areas [133]. 

## 5. Conclusions

Flax is a versatile crop that can acclimatize to several parts of the EU. It constitutes a source for a variety of industrial products while simultaneously having a high nutritional value that could strengthen any food system and enhance food security. The compounds in its seed oil can be used in medicine. It can be grown organically or under no-till regimes, and it can be grown with relatively low inputs depending on the soil and precipitation. Rather than being a “miracle crop”, flax exemplifies the goals of recent EU agricultural policymaking. Further studies should be conducted for policymakers within the EU to utilize the crop to its full potential in future agri-environmental strategies.

## Figures and Tables

**Table 1:** Flax fiber traits. TS stands for tensile strength and UTS for ultimate tensile strength. Data retrieved from Vaisey-Genser and Morris, 2003 [16].

Table	Value	Unit
Modulus	100	GPa
TM	27.6	GPa
Max diameter	600	μm
UTS	1100	MPa
Density	1.5	g/cm^3^
Max TS	1500	MPa
Max elongation at break	3.2	%

## Data Availability

The data presented in this study are openly available in Chand et al. (2020) [5], FAOSTAT [8], and USDA [41,51].
